# Multi-Dimensional Measurement of Antibody-Mediated Heterosubtypic Immunity to Influenza

**DOI:** 10.1371/journal.pone.0129858

**Published:** 2015-06-23

**Authors:** Jiong Wang, Shannon P. Hilchey, Ollivier Hyrien, Nelson Huertas, Sheldon Perry, Manojkumar Ramanunninair, Doris Bucher, Martin S. Zand

**Affiliations:** 1 Division of Nephrology, Department of Medicine and the Rochester Center for Biodefense Immune Modeling, University of Rochester Medical Center, Rochester, New York, United States of America; 2 Department of Biostatistics and Computational Biology, University of Rochester Medical Center, Rochester, New York, United States of America; 3 Rochester Center for Health Informatics, University of Rochester Medical Center, Rochester, New York, United States of America; 4 Department of Microbiology and Immunology, New York Medical College, Valhalla, New York, United States of America; Icahn School of Medicine at Mount Sinai, UNITED STATES

## Abstract

The human immune response to influenza vaccination depends in part on preexisting cross-reactive (heterosubtypic) immunity from previous infection by, and/or vaccination with, influenza strains that share antigenic determinants with the vaccine strains. However, current methods for assessing heterosubtypic antibody responses against influenza, including the hemagglutination-inhibition (HAI) assay and ELISA, are time and labor intensive, and require moderate amounts of serum and reagents. To address these issues we have developed a fluorescent multiplex assay, mPlex-Flu, that rapidly and simultaneously measures strain specific IgG, IgA, and IgM antibodies against influenza hemagglutinin (HA) from multiple viral strains. We cloned, expressed and purified HA proteins from 12 influenza strains, and coupled them to multiplex beads. Assay validation showed that minimal sample volumes (<5 μl of serum) were needed, and the assay had a linear response over a four Log_10_ range. The assay detected nanogram levels of anti-influenza specific antibodies, had high accuracy and reproducibility, with an average percentage coefficient of variation (%CV) of 9.06 for intra-assay and 12.94 for inter-assay variability. Pre- and post-intramuscular trivalent influenza vaccination levels of virus specific Ig were consistent with HAI titer and ELISA measurements. A significant advantage of the mPLEX-Flu assay over the HAI assay is the ability to perform antigenic cartography, determining the antigenic distances between influenza HA’s, without mathematical correction for HAI data issues. For validation we performed antigenic cartography on 14 different post-influenza infection ferret sera assayed against 12 different influenza HA’s. Results were in good agreement with a phylogenetic tree generated from hierarchical clustering of the genomic HA sequences. This is the first report of the use of a multiplex method for antigenic cartography using ferret sera. Overall, the mPlex-Flu assay provides a powerful tool to rapidly assess the influenza antibody repertoire in large populations and to study heterosubtypic immunity induced by influenza vaccination.

## Introduction

Annual immunization against influenza infection is one of the largest coordinated international public health efforts [1, 2]. Antibodies directed against the influenza surface hemagglutinin protein (HA) are a major source of protective immunity, blocking viral binding of the HA1 subunit to sialic acid expressed on the surface of target epithelial cells, and preventing viral entry [3]. Current flu vaccination strategies elicit protection primarily through the generation of long lasting, type-specific, neutralizing anti-HA antibodies. Influenza virus infection and vaccination both induce antibodies that bind to molecularly similar influenza subtypes, a phenomenon termed heterosubtypic immunity (HSI), and a major reason for the success of seasonal influenza vaccination [4, 5]. Vaccines containing HAs with small antigenic changes from the prior years’ influenza strains have a high probability of inducing memory B cells to class-switch and secrete IgG anti-HA antibodies. In contrast, pandemic strains are defined by low antigenic homology to prior vaccines and previously circulating influenza strains, and evade the human adaptive immune response [6–9].

Every individual has a different history of influenza vaccine and infection exposure, and thus has a unique pattern of heterosubtypic immunity. HSI can be quantified by measuring antibody reactivity against a large panel of influenza virus HAs from multiple strains. Because the immune response to any new influenza vaccine is a function of HSI, data regarding the extent of pre-vaccination HSI is crucial to assess the effectiveness of vaccination and population immunity. Current seasonal intramuscular inactivated influenza vaccine formulations contain HAs from three to four influenza viruses: one H3N2 and one H1N1 influenza A strain, and one or two influenza B strains [10]. The protective antibodies that arise from seasonal intramuscular influenza vaccination react against epitopes in the viral head region of the HA1 domain. If the vaccine strain contains HA1 sequences similar to those from prior vaccine or circulating strains, a robust and protective cross-reactive immune response occurs. In addition, many of the HA epitopes from different subtypes of influenza A viruses are homologous in the conserved HA2 stalk domain [11, 12]. Prior vaccination and/or influenza infection generally induces antibodies that also bind to the homologous stalk regions on the HA2 subunit, and confers some broadly cross-reactive immunity.

As different individuals exhibit varying degrees of antibody-mediated HSI both prior to and after vaccination, it is difficult to evaluate vaccine efficacy. Individuals with pre-existing HSI generally will have a strong memory B cell recall response to vaccination, and exhibit larger and more rapid antibody secretion response to vaccination with related influenza strains. The absence of preexisting HSI may result in a weaker response and less protective immunity [8]. As such, determining influenza vaccine efficacy requires that we know the pre-vaccine baseline pattern of HSI. This can be accomplished by multiple measurements of IgG, IgA, and IgM reactivity against a panel of HAs from multiple influenza strains. In addition, when new influenza strains emerge, (e.g. A/California/07/2009 H1N1 pdm), understanding the effects that pre-existing HSI has on the vaccine response to new viral strains becomes critical for vaccine selection. Identifying those subjects lacking cross reactive HSI, who may require both priming and a booster vaccination to induce protective immunity, may improve population immunity.

Current assays for measuring vaccine efficacy include the hemagglutination-inhibition (HAI) assay, enzyme-linked immunosorbent assay (ELISA), and to a lesser extent the microcytotoxicity assay [13–15]. The HAI assay measures the ability of post-immunization sera to prevent binding of the viral hemagglutinin to target sialic acid residues on the surface of red blood cells, causing RBC clumping. Although it was developed more than 70 years ago, the HAI assay is still the gold standard for serotyping influenza viruses, and determining antigenic relatedness among strains. Recently, however, the application of this assay has been complicated due to changes in the HA of H3N2 viruses, which have lost their capacity to agglutinate either chicken or turkey red blood cells, both of which are popular choices for use in HAI assays [16]. In addition, the HAI assay will not directly identify antibodies directed at the stalk region of influenza HA, which exhibit broad heterosubtypic reactivity among influenza strains and high efficacy at inhibiting influenza infection [17–20].

An alternative antibody binding assay is the ELISA [21], which identifies both anti-HA stem and sialic acid binding site antibodies. However, it requires significant amounts of serum and test antigens, is mono-specific for a selected recombinant HA protein, and requires multiple serial dilutions to accurately quantify anti-influenza strain specific antibodies. This makes the ELISA laborious, reagent intensive, and impractical for evaluation of population immunity from a large number of samples. A third assay also used to analyze vaccine responses is the microcytotoxicity assay [22], which measures actual inhibition of viral infection by mixing serum with single cycle infection viruses in the presence of target tissue culture cells. Each virus must be assayed individually, making the assay of reactivity against multiple viral strains in large numbers of samples or subjects are time consuming and difficult. All of these methods are also largely ignore the complexity of pre-vaccine HSI, making it difficult to judge vaccine efficacy as a function of pre-existing cross-reactive immune memory against multiple HAs.

Statistical methods for analyzing immune reactivity data are also used to select the strains to be included for seasonal vaccination [23]. Multi-dimensional data regarding serum antibody reactivity against multiple influenza strains is generally combined with antigenic cartography, a dimensional reduction technique that mathematically maps the similarity of viral proteins using an array of results quantitating antibody binding to a viral surface protein (e.g. HAI assay titers against 18 influenza strains)[24–26]. The result is a low-dimensional, usually bivariate diagram where the relative distances between points, representing influenza strains, is proportional to the total differences in antigenic reactivity. The gold standard for this type of analysis has been to use HAI assay data to test the cross-strain reactivity of influenza anti-sera produced from single strain viral infection or vaccination, from which antigenic similarity can be calculated [10, 24]. However, titer data from HAI assays have numerous issues requiring extensive mathematical modifications before analysis. HAI data is discontinuous, with the results having only 12 possible values from 1:10–1:5120. This property decreases the precision of antigenic cartography. In addition, HAI data requires several quantitative adjustments to account for titer values above or below the level of detection regarding viral strain antigenic similarity [23, 27, 28]. The precision of antigenic cartography and the simplicity of the computational methods could, however, be improved by using an assay that gives a continuous measure of anti-HA Ig binding.

To address these issues, and provide a more accurate and precise method of high-dimensional assessment o fboth HSI and viral antigenic similarity, we have developed and validated a fluorescent multiplex assay, the mPlex-Flu assay. The assay rapidly and simultaneously measures anti-influenza HA concentrations for IgG, IgA, and IgM against multiple influenza strains simultaneously over a 4-log_10_ range using only 5 μl of test sera. We report here its use for characterizing antibody reactivity against up to 12 influenza viruses HA simultaneously. Advantages of the mPlex-flu assay include a continuous linear output (as opposed to a discontinuous titer), a large range of accuracy, simultaneous measurements of reactivity against multiple HA’s from a single sample, greatly increased assay speed, and low inter- and intra-assay variability. To demonstrate these advantages, we have utilized this assay to analyze human vaccine responses in a cohort of healthy subjects prior to, and after vaccination with the intramuscular 2012 seasonal influenza vaccine. The total concentration of IgG, IgA, and IgM (sum total of binding activity against H1, H3, and B strains within the 2012 seasonal intramuscular influenza vaccine) assessed by mPlex-Flu assay were significantly correlated with HAI and ELISA measurements. We also report the use of the mPlex-Flu assay to evaluate HSI using ferret influenza virus strain specific anti-sera and strain specific monoclonal antibodies.

## Material and Methods

### Human Subjects Protection

This study was approved by the Research Subjects Review Board at the University of Rochester Medical Center (RSRB approval number RSRB00012232). Written informed consent was obtained from all participants, and kept on file per RSRB regulations. Research data were coded such that subjects could not be identified, directly or through linked identifiers, in compliance with the Department of Health and Human Services Regulations for the Protection of Human Subjects (45 CFR 46.101(b)(4)). Subject identification numbers were also randomly re-encoded for publication.

### Standard and Vaccine Study Serum

Standard curves correlating Ig concentration to Luminex immunofluorescence were generated using a standard serum mixture obtained by pooling sera from two subjects who had received the seasonal TIV vaccination (2013) and from one subject from our previous influenza vaccine study [29] who exhibited high titers of IgG, IgA, and IgM to all eight influenza hemagglutinins contained in our mPlex-flu assay. Total concentrations of IgG, IgA, and IgM from this standard serum were determined by standard serum immunoglobulin clinical assay of 7.25, 1.91, and 0.47 mg/ml, respectively. Vaccine study serum samples used for analysis by mPlex-Flu came from 10 healthy subjects enrolled in an influenza vaccine study who received the 2012 TIV intramuscular vaccine. Serum Ig concentrations were determined using traditional ELISA and HAI assays as previously described [29].

### Cloning and Expression of Full Length HAs

HA0 ORF sequences were amplified by two-step RT-PCR using Superscript III reverse transcriptase and Platinum Pfx DNA Polymerase and cloned into modified pFastBac CT-TOPO vector (Invitrogen, Grand Island, NY) from viral RNA isolations kindly provided by Drs. M. Ramanunninair and D. Bucher (New York Medical College, Valhalla, NY), which encode one H1 strain A/California/07/2009, three H3 strains A/Perth/16/2009, A/Victoria/210/2009, A/Victoria/361/2011, two B strains B/Brisbane/60/2008 (Victoria Lineage) B/Wisconsin/01/2010 (Yamagata Lineage) composited 2010–2013 TIV and two control H3 strains A/Port Chalmers/1/1973 and A/Hong Kong/1/1968, a total of 8 influenza viral strains (Table 1). Generated recombinant constructs were transformed into DH10Bac cells and recombinant bacmids were prepared using the PureLink highpure miniprep kit according to the manufacturer’s instructions (Invitrogen, Grand Island, NY). The recombinant baculoviruses were generated by transfection of *Spodoptera frugiperda* (Sf9) cells with the recombinant bacmids using Cellfectin II Reagent (Invitrogen, Grand Island, NY).

**Table 1:** Agglutination Activity of Influenza Vaccine Recombinant Hemagglutinins (rHAs).

	Strains	Gene Bank Accession No.	Agglutination activity (U/ug)	Code of Bio-Plex magnet-beads
H1N1	A/California/07/2009 ^a^ ^,^ ^b^ (Reassortant X-179A)	GQ214335.1	640	34
	A/South Carolina/1/1918 ^#^ (FR-692)	AF117241.1	ND	36
H3N2	A/Perth/16/2009 ^a^	GQ293081.1	<10	37
	A/Victoria/210/2009 ^a^ (Reassortant X-187)	HQ378745.1	320	43
	A/Victoria/361/2011 ^b^	KC306165.1**	160	28
	A/Texas50/2012 ^c^ (Reassortant X-223A)	KF752447.1 **	ND	62
	A/Hiroshima/52/2005 ^#^ ^d^ (FR-63)	EU283414.1	ND	64
	A/Port Chalmers/1/1973 ^d^	CY009348.1	2560	54
	A/Hong Kong/1/1968 ^d^	HM641178	2560	55
B	B/Brisbane/60/2008 ^a^ (Victoria Lineage)	CY115343.1	>5120	44
	B/Wisconsin/01/2010 ^b^ (Yamagata Lineage)	KC306166.1**	>5120	45
	B/Massachusetts/2/2/12 ^c^ (Reassortant BL-51B, Yamagata Lineage)	KF752446.1**	ND	65

^a^ The component of 2010–2011, and 2011–2012 TIV vaccine

^b^ The component of 2012–2013 TIV vaccine

^c^ The component of 2013–2014 TIV vaccine

^d^ The control H3N2 strains

** Submitted to gene bank by our lab

^#^ Recombinant protein provided by IRR

### Recombinant HA Expression, Purification, and Characterization

Each recombinant baculovirus was amplified in 3 passages, and then used to infect 250 ml of Sf9 cells (2.5×10^6^ cells/ml) in ESP921 medium (Expression Systems, Davis, CA) at an MOI of 1. After 18 hours, 5% Boost Additive (Expression Systems, Davis, CA) was added and cells were incubated for 5 days at 27°C shaking at 250 rpm. After 5 days the infected cells were harvested by centrifugation (5000g, 20 min) and cell pellets lysed with 25 ml xTractor Buffer (Clontech, Mountain View, CA) containing complete EDTA-free protease inhibitor. Lysates were further disrupted using a French press (Glen Mills, Clifton, NJ) at 2200 psi. The soluble lysate was harvested by centrifugation 15×10^3^ rpm (Beckman S1 rotor) at 4°C for 30 min and then incubated with 1 ml pre-equilibrated HisTALON resin (Clontech, Mountain View, CA) for 1 hour. The resin-supernatant mixture was loaded into glass 1.5 cm × 10 cm columns (Bio-rad, Hercules, CA), and retained resin was washed with 20 column volumes (*cv*) equilibration buffer (Clontech, Mountain View, CA) and 20 *cv* of Wash Buffer (equilibration buffer containing 10 mM imidazole), followed by 10 *cv* of Elution Buffer (containing 150 mM imidazole). The elution fractions were evaluated by NuPage 4–12% Bis-Tris gels (Invitrogen, Grand Island, NY) and Western-blot. Western-blot analysis was performed using rabbit anti-influenza strain/subtype specific polyclonal primary Abs (eEnzyme, Gaithersburg, MD) and detected using goat anti-rabbit horseradish peroxidase conjugated secondary Abs (Bio-Rad, Hercules, CA). Verified recombinant HA proteins were concentrated and desalted using Amicon Ultra 30K filters (Millipore, Billerica, MA). The purified HA proteins were evaluated by Hemagglutination assay [30], and digested by trypsin, Endo H, and PNGase F glycosidases [31] according to the manufacturer’s instructions (New England BioLabs, Ipswich, MA) to verify the characteristics of each recombinant HA. We also used several commercially obtained recombinant histidine tagged H1 influenza HAs: A/South Carolina/1/1918 (H1N1, IRR catalog No: FR-692), and A/Hiroshima/52/2005 (H3N2, IRR catalog No: FR-63) from Influenza Reagent Resource (IRR, Manassas, VA)

### Magnetic COOH Bead Coupling of Recombinant HA Proteins

Purified recombinant HA proteins for each influenza strain/subtype were covalently coupled to Bio-plex Pro Magnetic COOH Beads (Bio-Rad, Hercules, CA) using the Bio-Plex Amine Coupling Kit (Bio-Rad, Hercules, CA) (Table 1) according to the manufacture’s instructions. Purified HA proteins (12 μg) were individually bound to activated COOH beads (3.75×10^6^ beads per coupling reaction) for 2 hours in the dark separately, and unreactive sites on the beads surface blocked with 1% bovine serum albumin (BSA) in PBS for 30 min. Protein binding efficiency was tested with anti-HA subtype specific rabbit polyclonal Abs and a goat anti-rabbit PE-conjugated detection antibody (SouthernBiotech, Birmingham, AL). Coupled beads were counted using a hemocytometer and stored at 4°C in the dark.

### Monoclonal Antibodies, Ferret Antisera, and Secondary Antibodies

Anti-HA monoclonal antibodies and polyclonal anti-influenza strain ferret sera were obtained from the Influenza Reagent Resource (IRR, Manassas, VA) and specific sera are delineated in Tables 2 and 3. Phycoerythrin (PE) conjugated goat anti-human IgG (γ chain specific), IgA (α chain specific), and IgM (μ chain specific) secondary antibodies, PE conjugated goat anti-ferret IgG, and anti-mouse IgG secondary antibodies were obtained from SouthernBiotech, (Birmingham, Al).

**Table 2:** Ferret polyclonal anti-HA serum provide by the Influenza Research Resource (IRR).

	Catalog No	Induce antigen or virus	HAI Titer
H1N1	FR-359	A/California/07/2009	5120
	FR-388	A/Brisbane/59/2007	1280
	FR-288	A/Brisbane/59/2007	6250
H3N2	FR-446	A/Perth/16/2009	5120
	FR-646	A/Victoria/210/2009	5120
	FR-647	A/Victoria/210/2009	1250
	FR-1079	A/Victoria/361/2011	320
	FR-292	A/Hiroshima/52/2005	1250
B	FR-392	B/Brisbane/60/2008 ^a^	1280
	FR-810	B/Wisconsin/1/2010 ^b^	640
	FR-1265	B/Massachusetts/2/2012 ^b^	1280

^a^ Victoria Lineage Virus

^b^ Yamagata Lineage Virus

**Table 3:** Mouse monoclonal antibodies provide by the Influenza Research Resource (IRR) in this study.

	Catalog No	Induce antigen or virus
H1N1pdm	FR-506	A/California/04/2009
	FR-507	A/California/04/2009
H3N2	FR-553	A/Perth/16/2009
	FR-557	A/Perth/16/2009
	FR-1122	A/Victoria/361/11
	FR-1123	A/Victoria/361/11
B	FR-829	Type B (B2 and B4 blend)
	FR-1132	rHA1 of B/Wisconsin/1/2010
	FR-1133	rHA1 of B/Wisconsin/1/2010
	FR-1134	rHA1 of B/Wisconsin/1/2010
	FR-1135	rHA1 of B/Wisconsin/1/2010
	FR-1136	rHA1 of B/Wisconsin/1/2010

(All antibodies provided at a concentration of 500 μg/ml).

### mPlex-Flu Assay: Quantification of Anti-Influenza IgG, IgA, and IgM

The mPlex-Flu assay immunoglobulin quantification method was adapted from the method of Lai and coworkers [32]. Assays were performed in 96 well black-walled microtiter-plates (Millipore, Billerica, MA). Just prior to assay, the coupled beads were vortexed for 15 seconds and diluted to 50 beads of each bead region per μl and added at 25 μl beads per well. Standard serum and second antibody dilutions were selected based on a two-dimensional titration using human standard serum described above. Test sera were diluted at 1/5,000 and 1/10,000 for IgG antibody: 1/400 and 1/800 for IgA, and 1/50 and 1/100 for IgM assays (optimal identified dilutions). All serum dilutions and washes were performed using 1X PBS (pH 7.2) containing 0.1% BSA (MP Biomedical, LLC, France) and 0.1% Brij-35 (Thermo Scientific, Waltham, MA). Twenty-five μl of diluted test sera were added to the 25 μl of beads in each well, in duplicate, and incubated at room temperature for 2 hours on a rotary shaker (500 rpm) in the dark. Wells were then washed twice with 150 μl of wash buffer and 50 μl 1:400 diluted PE conjugated anti-human IgG (γ chain specific), IgA (α chain specific), or IgM (μ chain specific) specific secondary antibodies (SouthernBiotech, Birmingham, AL) were added to the appropriate wells and the plates incubated for 2 hours at room temperature on a rotary shaker (500 rpm) in the dark. After 3 additional washes, beads in each well were suspended with 100 μl Luminex driving solution (Luminex, Austin, TX) and analyzed on a Magpix multiplex reader (Luminex, Austin, TX), and results expressed as median fluorescence intensity (MFI).

To relate MFI to absolute concentrations of influenza strain-specific IgG, IgA, and IgM in the standard serum, we used the equivalence of absorbance method as published by Quataert, et al [33]. Specifically, known concentrations of total IgG, IgA, and IgM from a standard serum were serially diluted, and incubated with a bead region coupled with a goat anti-human IgG+IgA+IgM (H+L) polyclonal antibody (KLP, Gaithersburg, ML) to capture total immunoglobulin. Simultaneously, the same standard serum was incubated with the influenza specific HA coupled bead regions in seven 3-fold serial dilutions for IgG (1/1,000–1/729,000), IgA (1/50-1/36,450), and IgM (1/10-1/7,290). All groups were then detected using the same PE conjugated goat anti-human IgG, IgA, or IgM specific secondary antibody. We then fit a 5 parameter logistic (5 PL) standard curve of the MFI versus concentration of total IgG, IgA, or IgM generated from the IgG+IgA+IgM polyclonal antibody coupled bead set (see Statistical Methods section). The amount of HA specific immunoglobulin (IgA, IgG, IgM) in the standard serum was calculated by multiplying the Ig assay concentration determined from the assay (calculated by inverse regression from the standard curve) by the samples dilution factor. We then created a 5 PL logistic standard curve by estimated concentrations of standard serum for each individual HA specific IgG, IgA, and IgM antibody.

### Characteristics of the mPlex-Flu Assay

We defined the lower limit of detection (LLOD) as the mean plus two standard deviations of the assay blank (Luminex HA coupled bead incubated only with the secondary detection antibody). The upper limit of detection (ULOD) was defined as the highest value on the linear portion of the 5 parameter logistic curve. The lower and the upper limits of quantification (LLOQ and ULOQ) for each influenza HA and each Ig isotype were defined using the working range of the assay. They were determined using the Milliplex Analyst 5.1 software. Intra-assay variation was determined by testing 7 sera in triplicates from same assay. The percentage intra-coefficient of variation (CV) was calculated for each serum by dividing the standard deviation of the triplicate estimated concentrations by their respective empirical mean, and multiplying by 100. Such % intra-CVs were computed across the working range, is reported for each virus strain. Inter-assay variation was determined by running three independent assays of those sera in triplicates, and by calculating an inter-CV between the average estimated concentrations from each run. Mean % inter-CVs were computed by averaging intra-CV obtained over the working range.

The molecular specificity of the mPlex-Flu assay was determined by pre-adsorption of sera with strain-specific recombinant HA protein, as described previously by Lai et al [32]. Briefly, HA specific antibodies in a human post-vaccination serum sample that exhibited very high levels of anti-influenza IgG, IgA, and IgM to all of the HAs (1:1,000 dilution, within the ULOQ range) were pre-adsorbed by incubation with individual specific recombinant HAs (0.25 μg) for 1 hour [29]. The six vaccine strains tested were (A/California/07/2009, A/Perth/16/2009, A/Victoria/210/2009, A/Victoria/361/2011, B/Brisbane/60/2008 and B/Wisconsin/01/2010). Similarly Ferret antisera (using the IRR reported HAI titer as the dilution) were pre-adsorbed by incubation with individual specific recombinant HAs. The resulting blocked serum was assayed with an mPlex-Flu bead set containing HAs from 11 influenza viral strains, including the 6 vaccine strains used for the pre-adsorption assay described above, as well as two H3 control strains (A/Port Chalmers/1/1973 and A/Hong Kong/1/1968); two new TIV vaccine strains for 2013–2014: A/Texas/50/2012 (H3N2) and B/Massachusetts/2/2012 (B strain, Yamagata lineage); and an H1 control sub-strain bead set coupled with recombinant H1 HA from A/South Carolina/1918 (IRR, Manassas, VA, Cat N0.FR-692). Each sample was assayed in triplicate wells.

### ELISA and HAI Assays

ELISAs were performed to evaluate HA-specific IgG, IgA, and IgM levels as previously described [29], for comparison with the mPlex-Flu assay results. Briefly, for the ELISA 96 well ELISA plates were coated overnight with 150 μl/well of 1:200 diluted, unfractionated trivalent intramuscular seasonal influenza vaccine (Flulaval, 2011–2012 Formula, GSK), followed by washing, and incubation with serial dilutions of post-vaccine serum for 2 hours. Plates were then washed and a 1:500 dilution of Ig isotype specific (goat anti-human-IgG, IgA, or IgM) peroxidase-labeled antibody (KPL, Gaithersburg, MD) was added at 100 μl/well. Plates were incubated at 37°C for 1 hour, washed and developed using 1-Step Ultra TMB substrate (Pierce reagent kit, provided by Influenza Reagent Resource (IRR, Manassas, VA) using the provided protocol [29].

### Determination of Heterosubtypic Immunity (HSI) using the mPlex-Flu Assay

To evaluate HSI, we assayed influenza strain/subtype specific ferret polyclonal antisera and murine monoclonal antibodies directed against each individual HA (Tables 2 and 3). HA-coupled mPlex-Flu beads (1000 beads/25 μl) were incubated with 25 μl of ferret antisera diluted at the reported HAI titer, or 25 μl of HA-specific murine monoclonal antibodies (1:200 dilution) per well at room temperature for 2 hours. The bead sets included 12 specific HA coupled beads used for the ferret anti-sera pre-adsorption experiments described above, and recombinant HAs from A/Brisbane/59/2007 (H1N1, IRR, catalog number FR-65) and A/Hiroshima/52/2005 (H3N2, from IRR FR-63). Following the incubation, the beads were washed and PE-conjugated anti-ferret IgG or anti-mouse IgG specific antibody (1:400) was added at 50 μl per well and incubated for two hours. The MFI was then determined as above.

### Statistical and Computational Analysis

We described MFI as a function of the Ig concentration using the 5-parameter logistic curve:$F_{MFI}(C)=\theta_{0}+{\left(\frac{\theta_{3}}{1+{\left(\frac{C}{\theta_{2}}\right)}^{\theta_{1}}}\right)}^{\theta_{4}}$, where *C* is the Ig concentration and where *θ*_0_,…,*θ*_4_ are free parameters to be estimated. This model was fitted to the data (either total or virus HA strain-specific IgG from serially diluted sera) using the method of weighted least-squares. The same approach was used for IgA and IgM to calculate the concentrations of HA specific IgA and IgM. Ig concentrations were estimated by using inverse regression.

Statistical analysis and data visualization were performed using *Mathematica* (version 9.1 or 10; Wolfram Research, Inc.). Associations between the mPlex-Flu, HAI, and ELISA assays were analyzed using the Pearson’s linear correlation coefficient. The phylogenetic tree of 13 influenza segment 4 genes encoding HA proteins of the vaccine strains used in this study was generated from the Influenza Research Database website (www.fludb.org)[34]. The antigenic distance dendrogram was generated by unsupervised hierarchical clustering using the squared Euclidean distance measure. Cluster fusion level was determined based on average cluster dissimilarity. Metric multidimensional scaling was performed using custom *Mathematica* code and used to project 12-dimensional Euclidean distance relationships between influenza strain HAs onto two dimensional subspaces. The *Mathematica* code is available upon request.

## Results

### Properties of the Recombinant Influenza HA Proteins

We first examined the glycosylation patterns of the cloned, expressed and isolated HA proteins used in the mPLEX-Flu assay, as they were expressed in a baclovirus system. Proper glycosylation and secondary structure of the influenza HA is critical for its immunogenicity and biological activity. Recombinant HA (rHA) proteins prepared from different expression systems, such as human cell-lines and insect cells, have been shown to differ in their biological activity and ability to bind anti-HA antibodies due to differences in glycosylation patterns and protein folding from natively isolated virus [30]. In addition, it has been reported that rHA expressed by insect cells appeared to preserve both the antigenic properties and biological activity of HA proteins [31]. We therefore used a baclovirus system to express full-length HA0 proteins with sf9 insect cells from all eight influenza strains covered by the 2010–2013 influenza vaccines as well as two H3 control subtypes (Table 1).

Expressed rHA0 proteins were purified with Talon metal affinity resin with a purity of rHA0 proteins > 90% (S1 Fig). We first confirmed the molecular weights and antigenic sequences of these rHA0 proteins by western blot analysis (S2 Fig). In addition, as shown in S4 Fig, trypsin digestion of each rHA0 produced the expected HA1 and HA2 fragments. Gel-shift assays after digestion with PNG F or Endo H endoglycosidases (S3 Fig) confirmed that the expressed rHA0s were fully glycosylated. Lastly, the sialic acid binding activity of each rHA was assessed by turkey RBC aggregation (Table 1), confirming biological activity of the recombinant HAs. Three recombinant HA proteins from the H3 vaccine strains, A/Perth/16/2009, A/Victoria/210/2009, A/Victoria/361/2011 showed significantly reduced RBC agglutination activities compared with that of two older H3 strains (A/Port Chalmers/1/1973 and A/Hong Kong/1/1968) and other H1 influenza A and B strains HA proteins, despite similar glycosylation patterns (S3 Fig). This finding is consistent with results reported by other investigators that identified HA mutations in the sialic acid binding site region of the recent H3N2 viral strains, resulting in the attenuation of chicken and turkey RBC agglutination activity by these strains [16]. This loss of agglutination activity has complicated interpretation of traditional HAI assays utilizing the recently emergent H3N2 viral strains, but this is not an issue for the mPLEX-Flu assay.

### Detection of the influenza strain-specific IgG, IgA, and IgM

**Fig 1:**
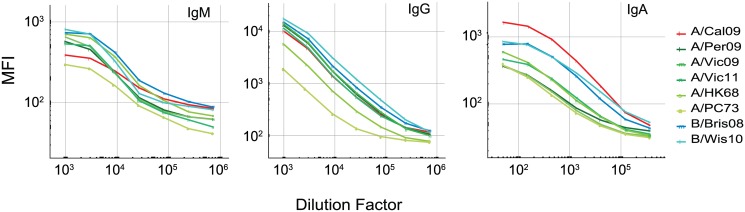
The mean fluorescence intensities (MFIs) generated from the binding of eight unique rHA-coupled beads (octoplex assay) with serially diluted human serum for determination of HA specific IgG, IgA, and IgM antibody amounts. A two-step Luminex assay was performed with recombinant hemagglutinins (rHA) of influenza vaccine related subtype strains: A/California/07/2009 (A/Cal09), A/Perth/16/2009 (A/Perth09), A/Victoria/210/2009 (A/Vic09), A/Victoria/361/2011 (A/Vic11), B/Brisbane/60/2008 (B/Bri08), B/Wisconsin/1/2010 (B/Wis10), B/Massachusetts/2/2012 (B/Mass12), A/Port Chalmers/1/1973 (A/P.C 73), A/Hong Kong/1/1968 (A/HK68) as described (see Methods). **(a)** Detection of human rHA specific IgG (seven 3-fold serial dilutions starting from 1:1000). **(b)** Detection of human rHA specific IgA (seven 3-fold serial dilutions starting from 1:50), and **(c)** Detection of human rHA specific IgM (seven 3-fold serial dilutions starting from 1:10). Serum dilutions for the different Ig isotypes were based on relative concentrations needed to remain within the 4-Log_10_ linear range of the mPlex-Flu assay.

The mPlex-flu assay presents an array of HAs to a small amount of serum, giving rise to the possibility that Ig species which cross-react to conserved epitopes across multiple HAs may bind to multiple beads. If a widely cross-reactive anti-HA antibody was present in small amounts, this may decrease the mPLEX-flu sensitivity for individual HA proteins. To assess if competition for antibodies with cross-reactive specificities would occur, we assayed antibody binding against individual beads (monoplex) and a combined mixture of 8 coupled bead sets (octoplex) by incubation with 3-fold dilutions of standard serum (Fig 1). The MFIs of the monoplex beads were nearly identical to those generated using the octoplex bead set (see S6 Fig) This result is consistent with minimal detectable interference when assaying multiple beads sets together simultaneously, compared to assaying the bead sets separately. This result is identical to similar studies of Luminex assays reported by others [32, 35].

As both IgA and IgM play important roles in the antibody-mediated immune response to influenza [36, 37], as such we also assessed the absolute amounts of HA specific IgG, IgA, and IgM using anti-human α-, γ- and μ- chain specific secondary antibodies (Fig 1b and 1c). Total serum concentrations of IgA and IgM are much lower than IgG, and we found substantially less HA specific IgA and IgM as compared to IgG. Thus, the serum dilutions for the assay were adjusted accordingly. Instead of the 1:1000 sera dilution used for IgG, the IgA required 1:50 and IgM 1:10 dilutions at the highest concentrations in the upper limit of the assay quantitation range. However, the assay performed well for all three isotypes, with a 4-Log_10_ linear range of sensitivity.

### Quantitation of Absolute Ig Levels

The primary goal of the mPlex-Flu assay is to precisely quantitate absolute immunoglobulin (Ig) levels, rather than titers, of multiple-strain specific anti-influenza Ig. To this end, we first generated a standard curve relating bead mean fluorescence intensity (MFI) to the absolute amount (ng/μl) of each Ig isotype directed against specific influenza HAs using the equivalence of absorbance method [33]. Equivalence of absorption measures total IgG, IgA, or IgM using serial dilutions of subject sera, rather than purified control Ig, captured by Luminex beads coated with a mouse anti-human IgG+IgA+IgM polyclonal capture antibody. The advantage of using subject serum, rather than purified human IgG, for example, is that this method controls for assay interference by other non-immunoglobulin proteins or compounds that may be present in test serum, minimizing inter-subject assay variance. The concentration of each Ig isotype is measured by an independent assay (see Methods), and the standard curve created by fitting a 5-parameter logistic curve to MFIs generated from a serial dilution of a reference serum for each isotype (IgG, IgA, and IgM), bound to the Luminex bead with the capture antibody (Shown in S5 Fig).

The concentrations for the IgG, IgA, and IgM specific antibodies against eight influenza stain HAs using the reference serum are shown in S1 Table. Thus the advantage of this method over the HAI assay is that the mPlex-Flu assay provides a continuous measure of HA-specific Ig for each isotype, rather than a discrete titer.

### mPlex-Flu Limits of Quantitation and Variability

The LLOQ and ULOQ of the mPlex-Flu assay are shown in Table 4. We found the LLOQ of the assay to be within the range of 0.06–16.24 ng/ml, and vary slightly depending on the detection viral strain: 0.09 ± 0.06 ng/ml antibody of anti-A/Perth/16/2009 to 0.34 ± 0.12 ng/ml anti-B/Wisconsin/1/2010 for IgG; 0.06 ± 0.06 ng/ml of anti-A/Port Chalmers/1/1973 to 0.39 ± 0.29 ng/ml B/Wisconsin/1/2010 antibody for IgA; and 0.14 ± 0.04 ng/ml of anti-A/California/07/2009 to 16.42 ± 0.08 ng/ml A/Hong Kong/1/1968 antibody for IgM. This is roughly 10 fold more sensitive than the traditional ELISA, which has a lower limit of detection of approximately 1.0 ng/ml for each Ig isotype. The mPlex-Flu assay showed a much larger working range than HAI or ELISA, covering a 4 to 5 log_10_ range with an ULOQ of 215–652 ng/ml for IgG, 24–841 ng/ml for IgA, and 40–236 ng/ml for IgM. For specific anti-influenza virus IgG antibodies, the assay was able to quantitate anti-A/California/07/2009 antibodies in the range of 0.14–215.3 ng/ml, anti-A/Perth/2009 0.09–221.85 ng/ml and anti-B/Brisbane/01/2008 0.30–632.85 ng/ml, with mean intra- and inter-assay percentage coefficients of variation of 9.06 and 12.94, respectively.

**Table 4:** Validation characteristics for each of the eight individual strains of HAs in the octoPlex-Flu assay.

	subtypes	LLOQ (ng/ml)	ULOQ (ng/ml)	R^2^ *	Intra-assay CV(%)	Inter-assay CV (%)
IgG	A/California/07/2009	0.14±0.06	215.30±41.12	1.000	4.93±2.19	6.34±4.06
	A/Perth/16/2009	0.09±0.06	221.85±29.97	1.000	6.47±6.86	5.91±4.44
	A/Victoria/210/2009	0.12±0.06	274.41±74.65	1.000	9.30±7.67	5.56±3.60
	A/Victoria/361/2011	0.14±0.07	321.53±20.26	1.000	5.13±1.71	5.34±3.51
	A/Port Chalmers/1/1973	0.09±0.07	52.57±17.89	0.997	12.50±5.02	7.59±4.15
	A/Hong Kong/1/1968	0.22±0.17	208.07±96.82	1.000	17.86±20.30	9.50±7.19
	B/Brisbane/60/2008	0.30±0.13	632.85±134.98	1.000	6.93±4.18	6.11±7.28
	B/Wisconsin/1/2010	0.34±0.12	652.82±169.78	1.000	6.76±4.80	5.69±5.20
IgA	A/California/07/2009	0.83±0.74	841.17±468.10	1.000	6.78±3.48	6.13±5.06
	A/Perth/16/2009	0.18±0.29	48.42±14.12	1.000	7.09±7.54	6.34±2.74
	A/Victoria/210/2009	0.11±0.12	27.98±16.30	1.000	10.80±4.23	4.14±3.22
	A/Victoria/361/2011	0.07±0.04	101.51±16.44	1.000	5.16±1.18	5.23±1.61
	A/Port Chalmers/1/1973	0.06±0.06	24.34±7.42	0.997	15.70±12.77	6.65±5.60
	A/Hong Kong/1/1968	0.81±1.30	43.21±83.58	1.000	7.39±6.92	6.14±4.06
	B/Brisbane/60/2008	0.28±0.04	284.67±67.95	1.000	7.65±4.86	17.54±9.38
	B/Wisconsin/1/2010	0.39±0.29	158.05±122.63	1.000	6.08±6.34	4.44±5.30
IgM	A/California/07/2009	0.14±0.04	168.76±19.88	0.999	8.34±9.94	7.74±5.83
	A/Perth/16/2009	0.29±0.43	117.67±27.80	0.984	8.82±9.66	37.31±39.82
	A/Victoria/210/2009	0.62±0.63	128.885±21.26	1.000	10.10±3.65	29.71±31.74
	A/Victoria/361/2011	0.24±0.08	236.88±57.81	1.000	11.59±11.80	7.73±7.12
	A/Port Chalmers/1/1973	0.34±0.54	40.86±25.61	0.994	11.37±12.79	38.19±25.68
	A/Hong Kong/1/1968	16.24±0.08	99.19±50.93	0.998	6.19±3.54	26.43±8.62
	B/Brisbane/60/2008	1.03±0.66	140.65±29.25	0.998	15.13±17.27	33.70±28.88
	B/Wisconsin/1/2010	0.19±0.04	156.72±82.48	0.998	9.37±3.96	20.99±10.02
Ave					9.06	12.94

* In the LLOQ to ULOQ range, the R^2^ values.

Overall, the assay also exhibited low intra- and inter-assay variation (Table 4). The maximum intra-assay CV for IgG is 0.0052 (B/Wisconsin/1/2010), 0.0258 for IgA (A/Port Chalmers/1/1973), and for 0.0257 for IgM (A/Perth/16/2009). The inter-assay CV from 3 independent assays showed a variation of 0.20 for IgG, 0.18 for IgA, and 0.26 for IgM. These data indicate that the mPlex-Flu assay is both highly accurate and reproducible.

### Assessment of Assay Specificity and Heterosubtypic Immunity (HSI)

For this study, we defined assay specificity as the residual non-specific binding of post-vaccine or post-infectious serum pre-adsorbed with a specific HA coated reagent bead. This functional definition is also linked to HSI, as pre-adsorption the HA from one strain would be expected to show reduced binding to other HAs with similar antigenic determinants. To determine the specificity of the mPlex-Flu assay, and its ability to detect HSI, we next assayed sera from which we had removed different strain specific Ig by pre-adsorption with individual purified rHAs. Pre-adsorption was performed on both human reference serum, as well as polyclonal ferret influenza strain/subtype specific anti-sera. We selected a human serum sample that displayed a very high concentration of multiple anti-influenza HA specific antibody species. The human reference sera were pre-adsorbed by incubation with 2012 TIV vaccine or individual rHAs, while the ferret sera was pre-adsorbed using the rHAs from the same strain/subtype used to vaccinate the ferret and generate the sera.

**Fig 2:**
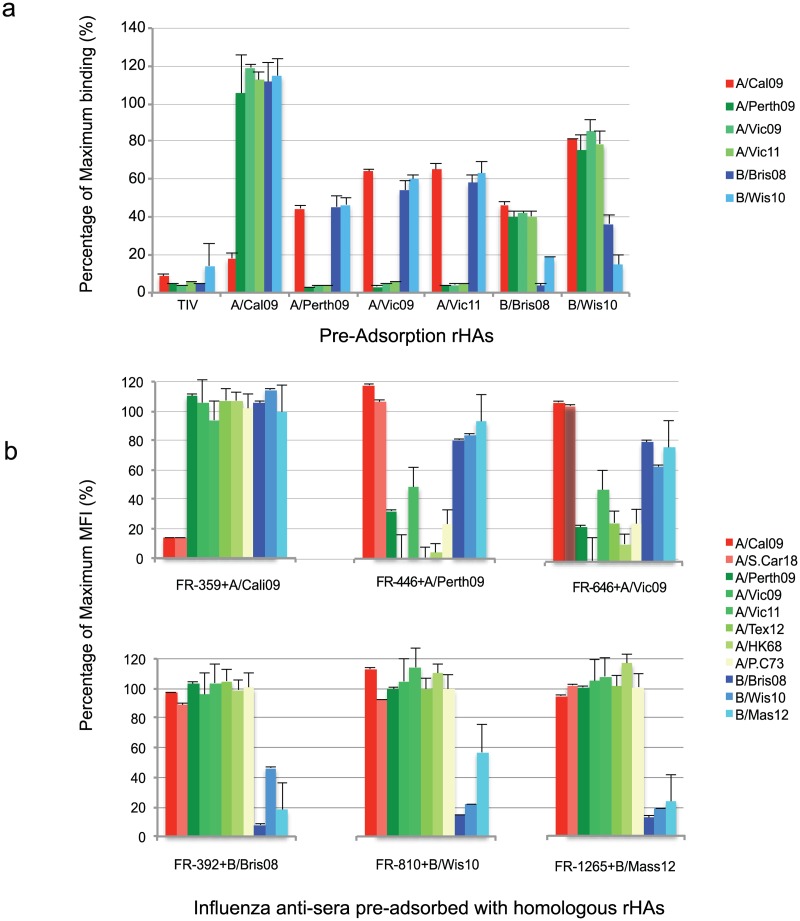
Evaluating the specificity of the mPlex-Flu assay by specific rHA protein pre-adsorption. **(a)** One human serum diluted to the upper limitation of quantitation (ULOQ) range of mPlex-Flu assay, was pre-adsorbed with rHA of A/California/07/2009 (A/Cal09), A/Perth/16/2009 (A/Perth09), A/Victoria/210/2009 (A/Vic09), A/Victoria/361/2011 (A/Vic11), B/Brisbane/60/2008 (B/Bri08), B/Wisconsin/1/2010 (B/Wis10) rHA proteins or the 2012 TIV. Each pre-adsorbed sera sample was directly compared to data generated using the mPLEX-Flu assay on the same sera sample prior to pre-adsorption. As expected, the maximum binding was observed with the un-absorbed serum. **(b)** Influenza virus specific ferret antisera pre-adsorbed with homologous rHA, and assayed using an 11 rHA-coupled mPlex-Flu bead set. Sera before or after pre-adsorption with rHAs homologous to the 11 bead sets were compared using the mPLEX-Flu assay. As with the human sera, the largest binding was seen with non-absorbed sera. Ferret sera designations: anti- A/California/07/2009 (FR-359), anti-A/Perth/16/2009 (FR-446) A/Victoria/210/2009 (FR-646), B/Brisbane/60/2008 (FR-392), B/Wisconsin/1/2010 (FR-810), anti- B/Massachusetts/2/2012 (FR-1265). Error bars represent the mean ± SD of % of maximum MFI performed in triplicate.

After pre-adsorption, each serum sample was assayed for HA binding using a set of reagent beads containing six different influenza vaccine strain HAs (A/California/07/2009, A/Perth/16/2009, A/Victoria/210/2009, A/Victoria/361/2011, B/Brisbane/60/2008, B/Wisconsin/1/2010, B/Massachusetts/2/2012). Pre-adsorption of the human high-titer reference serum with A/Cal09 rHA protein specifically inhibited more than 80% of the homologous antibody binding activity, while maximum binding MFI results were maintained to the influenza H3 subtypes and B strains (Fig 2a). When the human reference serum was pre-adsorbed with H3 homologous subtype rHAs, we observed a greater than 90% decrease in binding of all three anti-influenza H3 subtype antibodies, with a 40–50% inhibition of reactivity for the H1 subtype and B strains supporting a higher degree of heterosubtypic, cross-strain, immunity. However, between H3 subtype groups, including A/Perth/2009, A/Victoria/2009 and A/Victoria/361/11, pre-adsorption with H3 rHA proteins caused greater than a 90% decrease in binding of all three anti-influenza H3 subtype antibodies, indicating very strong cross-reactivity between these H3 subtypes. This was expected given that phylogenetic sequence comparisons indicate that these H3 subtypes have significant homology and share several B cell epitopes.

We next examined HSI by pre-adsorption of the reference serum with influenza B rHA. Pre-adsorption of the human reference serum with B/Brisbane/60/2008 rHA inhibited serum reactivity by 97% to homologous (Victoria lineage influenza B) viral HA, and 82% to the B/Wisconsin/1/2010 strain (Yamagata lineage influenza B, indicating heterosubtypic reactivity), while only 40% inhibition of serum reactivity to the A strain subtypes was observed. Similarly, analysis of the B/Wisconsin/1/2010 (Yamagata Lineage) rHA pre-adsorbed sera demonstrates that homologous HA reactivity was >80% inhibited, while B/Brisbane/60/2008 (Victoria lineage influenza B) heterosubtypic reactivity was >60% inhibited. In contrast, there was only 20% inhibition of the H1 and H3 A strain HA reactivity. These data suggest that the mPlex-Flu assay has remarkable specificity, permitting assessment of heterosubtypic anti-influenza antibodies and distinguishing between the major H1, H3, and B subtypes. In addition, this assay can be used to assess antigenic differences within the major subtypes that are concordant with results of protein sequence analysis.

The specificity of the mPlex-Flu assay also was cross-verified using single strain-specific, polyclonal, post-influenza vaccine ferret antisera pre-adsorbed with a panel of influenza strain specific recombinant HAs, including A/California/07/09, A/Perth/16/09, A/Victoria/210/09, B/Brisbane//60/08, B/Wisconsin/1/10, and B/Massachusetts/1/12. The pre-adsorbed ferret anti-sera was then studied with the mPlex-Flu assay using 11 strain-specific rHA coupled bead sets, which included the 8 rHAs described above, as well as two vaccine strains from the 2013 and 2014 TIV formulations, B/Massachusetts/2/2012 (reassortant strain X-51B), and A/Texas/50/2012 (reassortant strain X-223A, as well as A/South Carolina/1918). As shown in Fig 2b, ferret anti-A/California/07/09 (FR-359) serum pre-adsorbed with the homologous rHA A/Cal09 protein, decreased homologous reactivity by 80%, without significantly affecting H1 A/South Carolina/1/1918 reactivity, or antibody reactivity to H3 influenza A and B strain rHA. In addition, two of the anti-H3 ferret antisera generated by infection with anti-A/Perth/16/2009 (FR-446) and anti-A/Victoria/210/09 (FR-646), pre-adsorbed with specific homologous rHA protein resulted in a decrease in reactivity of the 6 tested H3 rHA-coupled beads by 60–95%. Similarly, the pre-adsorption of anti-Brisbane/60/2008 (FR-392), B/Wisconsin/01/2010 (FR-810), B/Massachusetts/2/2012 (FR-1265) ferret sera with homologous rHA proteins decreased serum antibody reactivity to B strain HA by 55%-92%.

Overall, these results with demonstrate that strain specific rHA protein pre-adsorption of human and ferret influenza-immune sera specifically blocks binding to homologous influenza strains. In addition, this serological assessment was also able to quantify HSI, and distinguish between rHA with accuracy reflective of influenza HA sequence and epitope homology.

### Evaluating Human Immunoglobulin Responses to a Multivalent Seasonal Influenza Vaccine

Our previous daily time-series study of trivalent intramuscular seasonal influenza vaccination demonstrated that most subjects exhibit a peak vaccine-strain specific antibody secreting cell response between days 6–10 post-vaccination [29]. This coincides with a rise in vaccine-strain specific anti-HA IgG levels, which then decrease slightly at day 21 post-vaccination [29]. However, select subjects begin to show an early IgG antibody response around day 7–8, which peaks around day 21 post-vaccination. To study this phenomenon more rigorously, we evaluated influenza vaccine specific IgG, IgA, and IgM antibody responses against the 2011–2012 TIV vaccination from a cohort of 10 healthy subjects. Sera were taken immediately before vaccination (day 0), and 8, 10, and 21 days post vaccination and were assayed against the 3 vaccine rHA, and an additional 5 related rHAs, and these results were directly compared to those generated using the traditional HAI assay and ELISA.

**Fig 3:**
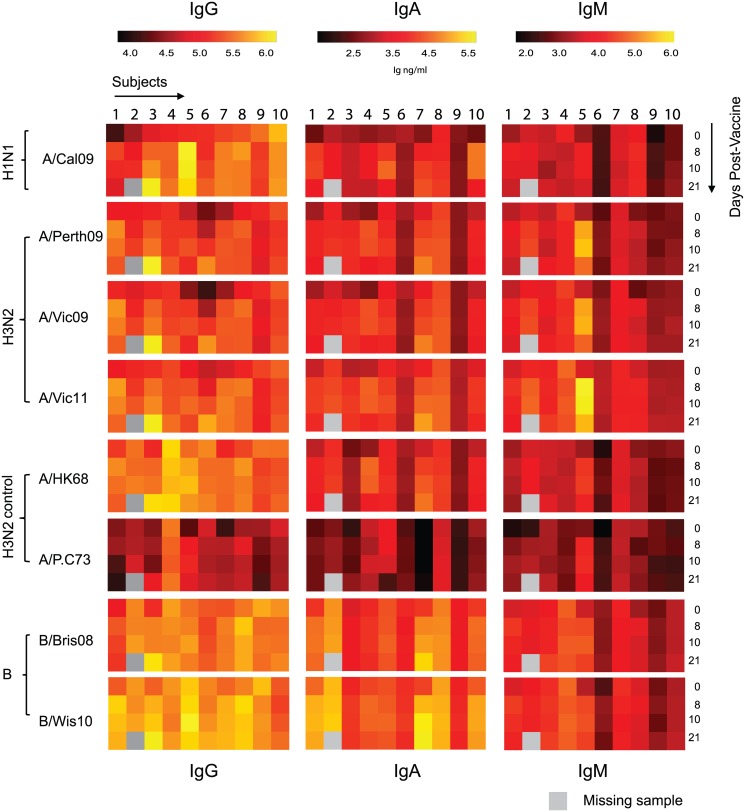
The specific antibody response to the 2012 TIV. We simultaneously measured the concentrations of vaccine-specific IgG, IgM, and IgA against the 2010, 2011, and 2012 seasonal TIV strains and control strains including: A/California/07/2009 (A/Cal09), A/Perth/16/2009 (A/Perth09), A/Victoria/210/2009 (A/Vic09), A/Victoria/361/2011 (A/Vic11), B/Brisbane/60/2008 (B/Bri08), B/Wisconsin/1/2010 (B/Wis10), A/Hong Kong/01/1968 (A/HK68), A/Port Chalmers/1/1973 (A/P.C73). Ten healthy subjects had serum samples taken in 2012 immediately prior to (day 0) and 8,10, 21 days post-vaccination with the 2012 TIV, followed by mPLEX-Flu assay. One subject had a missing serum sample on day 21.

Consistent with our previous observations [29], the mPlex-Flu analysis demonstrates that the seasonal TIV vaccine induced a strain-specific antibody response in most recipients (Fig 3). After intramuscular vaccination, most subjects displayed an increase in antibodies to all vaccine-specific subtypes with peak responses, varying by subject, on days 8, 10, or 21 post-vaccination. Only one subject lacked a significant vaccine-specific antibody response for all strains and all three Ig isotypes. Interestingly, this subject is one of two having the highest day 0 IgG concentration against A/California/07/09. However, neither subject showed an increase in IgG and IgM reactivity to any of the influenza rHA subtypes.

The mPLEX-Flu assay revealed a wide heterogeneity among the vaccine responses observed for different Ig isotypes, and among different strains. Several patterns were evident. For example, one subject had very high titers against the A/California/09 antibody, likely from by prior infection rather than vaccination [9]. However, we observed a moderate rise in vaccine-specific anti-A/California/04/2009 IgA despite minimal further increases in levels of anti-A/California/04/2009 IgG. Other heterogeneity was observed between strain responses that suggested a primary immune response. Subject 5 showed a substantial IgG antibody response to the H1N1 and B vaccine strains day 8 post-vaccination, but no response to the H3 vaccine-strains, and only a weak cross-reactive response to the H3 control strains at days 8 and 10 post-vaccination. In contrast, this subject had a significant IgM response to the H3 vaccine strains, but still with few heterosubtypic responses to the H3N2 control strains, suggesting a primary immune response to H3. Even with this smaller sample size, it is evident that vaccine-specific responses do not necessarily correlate between Ig isotypes. For example, a strong IgA response was observed in subject 7 by day 10 and 21 specifically to B strain virus, without a corresponding increase in vaccine specific IgM and IgG levels. Others (subject 3) demonstrate simultaneous and strong IgG responses (>15 times increase over day 0) for all strains we tested. Thus, measurement of HSI along with vaccine-specific responses allows for a comprehensive assessment of complex multiple antigen vaccine responses.

### Correlation with ELISA and HAI Assays

**Fig 4:**
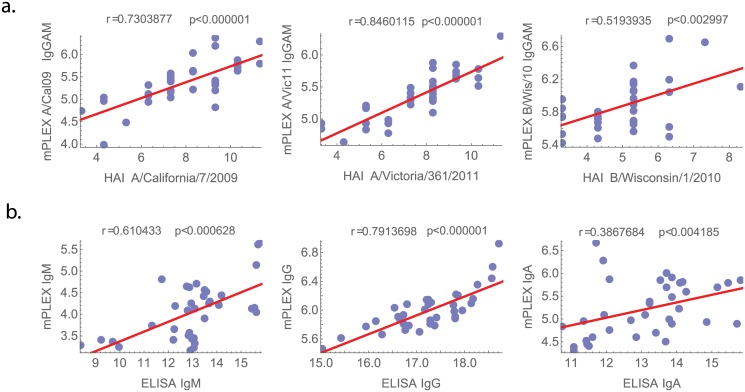
Correlation of mPlex-Flu assay with traditional ELISA and HAI assays. A comparison of the results of anti-vaccine specific antibody isotype levels (IgG+IgA+IgM) using the mPlex-Flu assay to HAI assay titers, and ELISA results for IgM, IgG, and IgA concentrations of A/California/07/09, A/Victoria/361/2011 and B/Wisconsin/1/2010 reactivity using the TIV vaccine antigens directly.

We measured the correlation between data generated using standard methods, the HAI assay and ELISA, with those generated by the mPlex-Flu assay using Pearson’s correlation coefficients (Fig 4). To achieve an accurate comparison between the HAI, which measures inhibition of sialic acid binding by all Ig isotypes combined, and the mPLEX-flu data, we summed the mPLEX-flu results for individual isotypes and compared this aggregate measure with the HAI titer. The sum of the mPlex-Flu measurements (IgG+IgA+IgM) for the vaccine specific antibody isotypes was significantly correlated with HAI titer results (the correlation coefficient values r > 0.5, Fig 4). Similarly, the results obtained by ELISA for IgM, IgG, and IgA concentrations of A/California/07/09, A/Victoria/361/2011 and B/Wisconsin/1/2010 using the TIV vaccine antigens directly, were significantly correlated with that obtained from the mPlex-Flu assay (p <0.003 in all cases) (Fig 4).

### Antigenic Profiles of Influenza HAs Assessed by mPlex-Flu Assay

**Fig 5:**
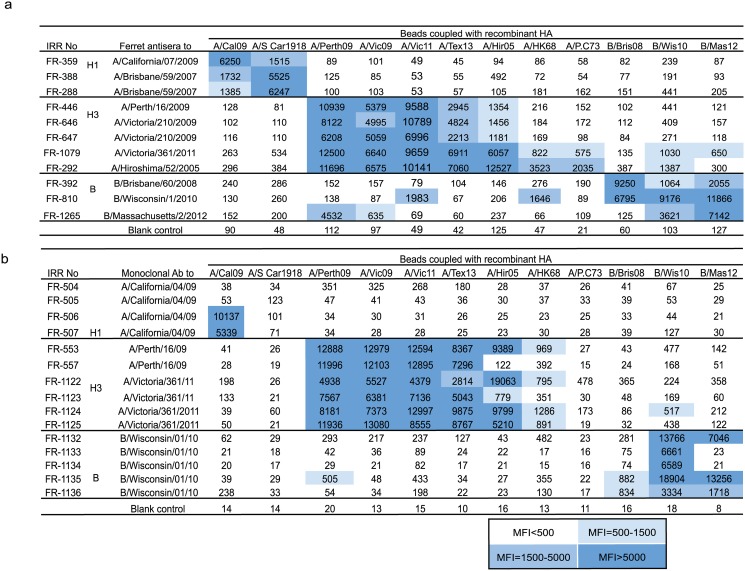
The binding reaction (MFI) of the mPlex-Flu assay using influenza ferret virus anti-serum and anti-influenza monoclonal antibodies. A panel of 12 rHA coupled-beads: A/California/07/2009 (A/Cal09) and A/South Carolina/1/1918 (A/S Car1918) H1 substrains, A/Perth/16/2009 (A/Perth09), A/Victoria/210/2009 (A/Vic09), A/Victoria/361/2011 (A/Vic11), A/Texas/50/2012 (A/Tex13), and A/Hiroshima/2005 (A/Hir05) A/Hong Kong/01/1968 (A/HK68) and A/Port Chalmers/1/1973 (A/P.C73) H3 substrains, B/Brisbane/60/2008 (B/Bri08), B/Wisconsin/1/2010 (B/Wis10), B/Massachusetts/2/2012 (B/Mass12) B strains, was used to measure antibody levels from; **(a)** post-infectious ferret antisera of influenza vaccine associated strains obtained from the Influenza Reagent Resource (IRR). Shown are the MFIs generated from the twelve-bead set assay utilizing dilutions based on the HAI titers provided by IRR. **(b)** The MFIs generated using mouse monoclonal antibodies directed against specific rHAs or whole influenza provided by the IRR. The concentration of all monoclonal antibodies in the assays is 2.5 μg/ml (1:200 dilution of 500 μg/ml). The MFI represents the mean of triplicates measurements.

We also explored the ability of the mPlex-flu assay to rapidly characterize the antigenic similarity between influenza HAs from disparate viral strains by antigenic cartography. Given that the mPlex-Flu assay can simultaneously measure reactivity against multiple influenza strains with a continuous quantitative measure, high sensitivity, and a 4 log_10_ range, we were interested to know how it would perform for rapid assessment of antigenic similarity among multiple disparate influenza strains. We tested a panel of ferret anti-influenza sera (Table 2) and murine monoclonal antibodies derived from single strain influenza rHA vaccinations (Table 3) against 12 rHA using the mPlex-Flu assay. This included rHAs from all eight influenza vaccine strains covered by the 2010–2014 TIV vaccines (A/California/07/2009, A/Perth/16/2009, A/Victoria/210/2009, A/Victoria/361/2011, A/Texas/50/2012, B/Brisbane/60/2008, B/Wisconsin/1/2010, B/Massachusetts/2/2012), three more temporally distant H3 control strains (A/Port Chalmers /1973, A/Hong Kong/1968 and A/Hiroshima/2005), and one temporally distant H1 control strain (A/South Carolina/1/1918). The results are shown in Fig 5. We found that A/California/07/09 rHA was strongly bound by homologous post-influenza vaccine ferret antisera (FR-359) but only weakly by H1 subtype (H1 strain) anti-A/Brisbane/59/2007 sera (FR-388 and FR-288), reflecting the antigenic shift of this pandemic virus. In contrast, the historical H1 control strain A/South Carolina/1/1918 showed stronger binding by the anti-A/Brisbane/59/2007 sera than anti-A/California/07/2009 post-infectious, suggesting that the antigenicity of A/South Carolina/1/1918 is closer to A/Brisbane/59/2007 than A/California/07/2009, at least in the context of primary influenza infection.

We next examined the antigenic similarity between H3 strains. Ferret serum from infection with H3 strains that appeared within the last 5 years showed strong cross-reactivity, but only weak reactivity against H3 hemagglutinins from H3 strains isolated over 40 years ago. Specifically, the rHA from the A/ Perth/2009, A/Victoria/210/2009 and A/Victoria/361/2011 strains strongly reacted to all H3 ferret homologous and heterosubtypic antisera, as well as several H3-specific monoclonal antibodies, suggesting that these three H3 HAs are quite antigenically similar. However, sera from ferrets vaccinated against the newer influenza strains displayed much lower reactivity against rHAs from older H3 strains, including A/Hong Kong/68 and A/Port Chalmers/73. Interestingly, post-infectious A/Perth/16/2009 and A/Victoria/210/2009 ferret sera showed decreased binding to the H3 strain for the recent 2013–2014 TIV vaccine H3 strain A/Texas/01/2012 compared to anti-A/Victoria/361/2011 ferret sera, reflecting antigenic evolution of the more recent H3 influenza A viruses. Similar observations were found for reactivity against influenza B strains of B/Brisbane/2008, B/Wisconsin/2010 and the recently emergent vaccine strain B/Massachusetts/2/2012 (Yamagata lineage). Vaccine strain-homologous sera demonstrated high MFI’s with homologous rHA, and relatively weak reactivity for sera obtained from heterologous vaccine strains.

**Fig 6:**
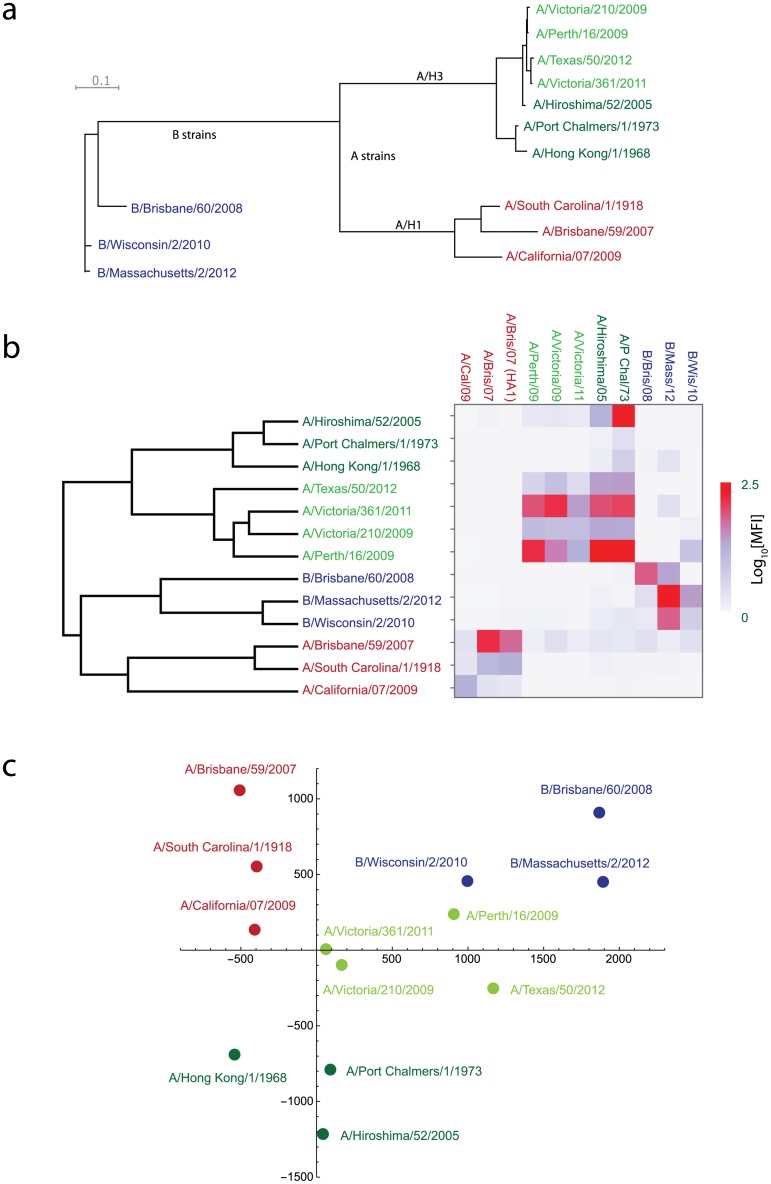
The phylogeny and antigenic cartography of a panel of HAs from influenza vaccine strain components within the 2012–2014 TIVs. **(a)** The phylogenic tree of HA genes of the influenza vaccine strains. **(b)** Heat map and dendrogram of the mPlex-Flu assay results using post-influenza infection ferret sera. Rows indicate the strain that the ferret was infected with to generate the strain-reactive serum, and columns indicate the recombinant influenza HA in the bead panel. Influenza strains are color coded based on the hierarchical clustering results, which were performed using a squared Euclidean distance measurement and cluster fusion level determined by the average cluster similarity metric. Note the segregation of the H3 strains by epoch (pre-2005 and post-2009). **(c)** Antigenic cartography generated with mPlex-Flu assay data. Relative antigenic distances are proportional to the distance between strains, reduced from 12- to 2-dimensions.

Summaries of the antigenic differences between the influenza strains, calculated from the mPlex-Flu data and displayed by hierarchical clustering and with metric multidimensional scaling, are shown in Fig 6. Note the distinct clusters of H1, H3, and B strains. The multi-dimensional scaling projection also highlights the relative antigenic similarity between the H1 strains A/California/07/2009 and the A/South Carolina/1/1918. In addition, hierarchical clustering based on the mPlex-Flu data shows two clear sub-clusters of H3 strains, with older strains of A/Hong Kong/1/1968, A/Port Chalmers/1/1973, and A/Hiroshima/2005 separated from newer strains of A/Victoria/210/2009, A/Perth/1/2009, A/Victoria/361/2011, and A/Texas/50/2012. Importantly, the mPlex-Flu assay was able to rapidly distinguish between two disparate Victoria and Yamagata lineages of B strain, showing that the results generated from the mPlex-Flu assay are strongly correlated with the antigenic sequence similarity of the HAs based on the genetic variance between each subtype (Fig 6).

## Discussion

An individual’s immune response to the seasonal influenza vaccine is dependent upon three factors: (i) cross-reactive (heterosubtypic) immunity (HSI) to the new seasonal influenza strains arising from previous vaccination against, or infection by, antigenically related influenza strains; (ii) the immunocompetence of the recipient; and (iii) the antigenic similarity of the actual circulating viral strains to those contained within the vaccine [2]. This heterogeneity makes it difficult to judge the degree of vaccine-induced change in the anti-HA immune repertoire in a simple manner, as human vaccine recipients each start with different underlying levels of HSI and immunocompetence. Adequately measuring HSI requires testing antibody reactivity against many influenza hemagglutinins, which is both time and labor consuming using current HAI assays and ELISA. To address this issue, we developed the mPlex-Flu assay, which simultaneously measures antibody reactivity against multiple influenza HAs in a large number of serum samples over a 4-Log_10_ range with high specificity and low intra- and inter-assay variability.

The major advantages of the mPlex-Flu assay are the ability to precisely quantitate multiple influenza antibody reactivities simultaneously in a large number of samples utilizing a very small sample volume, its continuous readout, a high specificity with low intra- and inter-assay variability, and an ability to detect very low levels of anti-HA Ig within a broad 4 Log_10_ range of detection. One benefit of the large range of detection is that the mPlex-Flu assay only requires 2 dilutions for sample measurements to fall within the linear range of the assay, as opposed to the ~12 dilutions required for the HAI assay. The mPlex-Flu assay is thus able to rapidly measure HSI in a large population in a timely and accurate manner, and allows study of the effect of pre-vaccine anti-influenza antibody levels on human influenza vaccine efficacy [5, 38–40]. This is a significant improvement in both quantitation of anti-influenza Ig activity, as well as high-throughput, multi-dimensional immune responses.

The results generated using the mPlex-Flu assay are highly correlated with traditional ELISA and HAI assays. However, the mPLEX-Flu assay can be performed much more rapidly and with a higher throughput, which will aid studies of influenza vaccine responses with large numbers of subjects and samples. Testing 9 subjects with 4 time points and 3 Ig isotypes against 6 influenza strains (Fig 5) took only a day. In contrast, recently published study measured HAI titers of 69 individuals pre- and post- influenza vaccination over 6 years against 35 influenza strains, for a total of ~16,000 individual HAI titrations with ~12 dilutions per titration [41]. If the mPLEX-Flu assay were used to analyze these samples, quantitation of 35 strain-specific IgG antibodies in the ~800 serum samples could be performed in approximately three weeks.

Another novel feature of the assay is its adaptability. For example, comparing results before and after blocking of the HA binding site with sialated biotin would mimic the functional nature of the HAI assay, permitting quantitation of the fraction of antibodies capable of blocking influenza virus binding to host cells. In addition, measurements using bead sets with native HA along with chimeric HA coated beads containing exotic HA1 head regions spliced with common HA2 stem regions will permit rapid and direct quantitation of the percentage of head versus stem binding antibodies in a single assay. Anti-stem antibodies have been recognized as a major factor in the development of broad HSI [18, 42, 43], but it is still unclear how much the broadly reactive stalk antibodies contribute to HSI. This work is currently underway in our laboratory.

An important application of the mPLEX-Flu assay is to improve analysis of the antigenic distance between different influenza strains. At present, the antigenicity of influenza virus HA proteins is determined by HAI assay, testing virus isolates against a panel of post-influenza infection ferret antisera with a mono-specific HAI assay for each viral strain. The data are then analyzed with multidimensional scaling, referred to as antigenic cartography [10, 24], where the distance between each HA represents the relative antigenic distance between the pair. Thus the precision of this analysis depends on the number of gradations available to specify antibody reactivity. The HAI assay relies on serial dilutions of subject serum (1:2, 4, 8, 16, 32, 64, 128, 256, 512, 1024, 2048, 4096), and thus has only 12 discontinuous readouts, which makes it essentially categorical. Also, the throughput of the HAI assay is low, making simultaneous measurement of large numbers of samples against large numbers of HA very difficult, and often resulting in missing data due to availability of enough serum. In addition, the HAI assay results require complex processing to adjust for results above or below the limit of detection. In contrast, the mPlex-Flu assay provides a continuous result for the vaccine specific Ig level (ng/ml) over a 4-log_10_ range. This distinction becomes critical when defining the antigenic “distance” between influenza strains using immune sera and antigenic cartography, multidimensional scaling, or other clustering methods, especially when values below 1:20 dilution must be imputed rather than measured [23, 28, 44, 45]. For example, a change in HAI titer from 2048 to 4096 results in a very large increment in the antigenic distance, even if the actual antibody change is much smaller in absolute concentration. Better definition of functional antigenic differences between viral strains is essential when quantitative measures of antigenic similarity between viral strains (e.g. HAI, ELISA, mPLEX-Flu) are used to select influenza strains for the seasonal vaccine [23, 41, 46, 47]. Thus, application of the mPlex-Flu assay will significantly enhance the accuracy of antigenic distance predictions for vaccine selection, as well as simplify the computational methods. Further large-scale studies will be needed to assess the precision of this method.

We have also reported the application of the mPlex-Flu assay to examine the development of influenza immunity in human subjects. Given the normal human population immunity heterogeneity, we would anticipate differences in vaccine responses due to genetic variation, prior viral exposure, and vaccination, as well as general immunocompetence of each individual. Indeed, in our study we did not find any subjects displaying a similar multi-dimensional pattern of antibody responses to 8 influenza substrains after intramuscular vaccination with the 2012 TIV. In the face of such individual variation, our mPlex-Flu assay allows characterization of an individual’s unique antibody repertoire. These results could then be correlated to the subject’s medical history, including their level of immunocompetence, influenza infection/vaccine history and other factors to improve vaccine strategies. Moreover, beyond individual vaccine responses, applying the mPlex-Flu assay to a large population will provide a multidimensional dataset mapping influenza immunity and post-vaccine antibody responses within the population as a whole. Such large-scale population studies have been significantly limited in scope in the past due to the cost and labor necessitated by traditional assays. The mPlex-Flu assay would enhance the development and application of mathematical models to predict population immunity and the population antibody repertoire.

The mPlex-flu assay will also improve our ability to quantify the influence of pre-vaccine HSI in the evaluation of human influenza vaccine efficacy [5, 38–40]. Most adults have a spectrum of prior influenza infection and vaccination, and thus have pre-vaccine HSI to strains with antigenic similarity the vaccine strains. The efficacy of vaccination has previously been defined by difference between the pre- and post-vaccine strain specific HAI titers to the vaccine strains. However, subjects who have weakly cross-reactive anti-HA antibodies, below the level of detection for HAI, will respond to the vaccine with more rapidity and more robust vaccine-specific IgG antibody levels [48]. The mPLEX-flu assay can rapidly detect such reactivity, pre- or post-vaccination, and this information could then be used to determine vaccine efficacy under a variety of immune conditions (naïve, with IgM reactivity, with weak IgG reactivity, with higher or graded IgG immunity). Another advantage of the assay is that pre-existing immune memory to other vaccine antigens (measles, rubella, hepatitis B surface antigen, tetanus) could be measured in the assay to simultaneously assess overall immune competence in populations with immune deficiencies or who are immunosuppressed.

A few caveats apply to the form of the mPLEX assay reported here. In this manuscript, we used the entire HA protein, including the transmembrane region, without a trimerization domain. The results presented may have two issues: underestimation of antibodies directed at the influenza HA stem, and reactivity against the short transmembrane domain giving a false elevation in anti-HA concentrations. These issues can be obviated by engineering recombinant HA lacking the transmembrane portion of the protein, and containing a trimerization domain [17]. This work is currently being performed in our laboratory. Additionally, cross-absorption, the binding of low levels of IgG to multiple HA proteins reducing the ability to detect the presence of that antibody, did not occur in our experiments. However, careful validation will be necessary as we add more HA strains to simultaneous measurements. Another potential limitation is the speed at which new influenza strain hemagglutinins can be cloned, expressed, and coupled to multiplex beads. In our hands, this takes approximately 4 weeks from cloning to protein purification and bead coupling, which would be sufficient time to add new vaccine or circulating viral strains to an existing assay panel.

Finally, we did not directly compare the mPlex-Flu assay to the recently described protein microarray methods [49–51]. These methods micro-print an array of HA proteins onto regions of a grid on a small slide, allowing simultaneous measurement of levels of anti-HA immunoglobulins to be quantified. Several important differences exist between the mPLEX-Flu assay and the micro-array methods. First, the HA’s in the mPLEX-Flu assay are covalently coupled to the bead at the carboxy terminus, minimizing the possibility of conformational changes which can occur with adsorbed proteins that may lead to false positive antibody binding. Secondly, the linear range of protein microarray assays has been reported to be ~2-logs [49–51], which is far below the 4.5 log range observed with the mPLEX-Flu assay. Additionally, microarray assays require expensive and specialized equipment, including a micro-printer and specialized scanner. In contrast, the mPLEX-Flu assay can be performed on more widely available micro-bead readers or standard low-dimensional flow-cytometers. The mPLEX-Flu method is also more flexible, allowing custom panels of reagent beads to be rapidly and easily combined for specific assay requirements, without reprinting the entire panel.

In summary, the mPlex-Flu assay appears to be a promising and powerful tool to accurately and rapidly evaluate preexisting heterosubtypic immunity, determine the antigenic similarity of influenza strains, and should significantly aid in future influenza vaccine strain selection.

## Supporting Information

S1 Fig Purified recombinant HA (rHA) Proteins of Influenza vaccine related strains. The Whole HA0 genes of influenza vaccine associated strains were expressed by pFastBac CH-TOPO vector (Invitrogen) that harbors a 6 His-tagged at the C-terminal. The rHAs were purified with HisTALON resin (Clontech) and concentrated with Amicon Ultra 30K filters (Millipore). The purified rHAs were separated and analyzed by 4–12% Bis-Tris NuPage gels (Invitrogen).
(EPS)

S2 Fig The antigenic characteristics of influenza vaccine rHAs determined by Western Blot. Purified recombinant HAs were separated on NuPage 4–12% Bis-Tris gels (Invitrogen) and transferred to Nitrocellulose membrane (0.45μm, Bio-Rad). After the membrane was blocked with 5% milk in TBST buffer (500 mM NaCl, 20 mM pH 7.5 Tris—HCl, 0.05% Tween-20)) for 30min at room temperature, it was detected with polyclonal influenza subtype specific antibodies (eEnzyme) against HA of different influenza vaccine strains at final dilution of 1:500. **(a)** Anti-HA of A/California/04/2009 (IA-01SW-0100), **(b)** Anti-HA of H3N2 (IA-PAN4-0100), **(c)** Anti-HA of Influenza B (IA-032-0100) **(d)** and the human positive reference serum at a 1:3000 dilution, overnight at 4°C. After washing with TBST 3 times, peroxidase conjugated goat anti-Rabbit IgG Fc fragment or goat anti-human IgG (H+L) secondary antibodies (Jackson Immuno) were added (1:5000 dilution) and incubated at room temperature for 1 hour. The resultant gel images was analyzed using the Bio-Rad ChemiDoc XRS+ luminescence reader and associated software.
(EPS)

S3 Fig Purified rHA proteins treated with PNGase F and Endoase H endoglycosidases. Recombinant HA proteins 20 μl 100 ng/μl were treated with 1 μl of 500 U/μl Endo H (NEB, Ipswich, MA), or 1 ul of 500 U/μl PNGase F (NEB, Ipswich, MA) at 37°C for 30 minutes, and then reactions were stopped and samples loaded on the 4–12% Bis-Tris SDS-PAGE gel. The observed decrease in the molecular weights of proteins after treatment demonstrates that the baculovirus expression system generates glycosolated rHA poteins.
(EPS)

S4 Fig Trypsin treatment of rHA proteins to generate HA1 and HA2 fragments. Recombinant HA proteins (20 μl at a concentration of 100ng/μl) were treated with 5 μl of 0.1 μg/μl Trypsin (NEB) on ice for 30 minutes, then the reactions were stopped by addition of 6 μl of sample buffer, and samples loaded on the 4–12% Bis-Tris SDS-PAGE gel.
(EPS)

S5 Fig Standard curve generated from the human reference serum using the Luminex assay. For estimation of weight-based Abs to influenza vaccine strains with standard serum, STD01, made by our lab. A bead region coupled with goat anti-human IgG+IgA+IgM polyclonal antibody was used to capture all three immunoglobulin subtypes, Total individual subtypes within the standard serum were detected using PE conjugated anti-human IgG, IgA or IgM specific second antibodies. The resultant MFIs were correlated with the known concentrations of total IgG, IgA, and IgM to create a standard curve by fitting the data to with a 5 parameter logistic (5 PL) non-linear regression model (F(x) = A + (D/(1+(X/C)^B)^E)) for **(a)** IgG; **(b)** IgA and **(c)** IgM Abs.
(EPS)

S6 Fig MFI generated from simultaneous HA monoplex and octoplex assays with the same serum. For comparison of mPlex-Flu assay monoplex and octoplex, we performed assays at simultaneously with identical conditions with only one bead set (monoplex) or all eight beads sets (octoplex) at the same time, and compared the MFIs generated from; **(a)** IgA of B/Brisbane/60/2008; **(b)** IgA of B/Wisconsin/01/2010; **(c)** IgG of B/Brisbane/06/2008; **(d)** IgG of B/Wisconsin/01/2010.
(EPS)

S1 Table The quantitation of specific antibodies against influenza vaccine associated subtypes rHAs within the STD01 standard serum generated by our lab. A/California/07/2009 (A/Cal09), A/Perth/16/2009 (A/Perth09), A/Victoria/210/2009 (A/Vic09), A/Victoria/361/2011 (A/Vic11), B/Brisbane/60/2008 (B/Bri08), B/Wisconsin/1/2010 (B/Wis10), A/Hong Kong/01/1968 (A/HK68) and A/Port Chalmers/1/1973 (A/P.C73).
(DOCX)
