# Supplementary material for: Multi-Dimensional Measurement of Antibody-Mediated Heterosubtypic Immunity to Influenza
Source: PLoS One. 2015 Jun 23;10(6):e0129858. doi: 10.1371/journal.pone.0129858 (PMC4478018; doi:10.1371/journal.pone.0129858)
Supplement: S1 Table — A/California/07/2009 (A/Cal09), A/Perth/16/2009 (A/Perth09), A/Victoria/210/2009 (A/Vic09), A/Victoria/361/2011 (A/Vic11), B/Brisbane/60/2008 (B/Bri08), B/Wisconsin/1/2010 (B/Wis10), A/Hong Kong/01/1968 (A/HK68) and A/Port Chalmers/1/1973 (A/P.C73). (DOCX) [file pone.0129858.s007.docx]

| HAs  (Influenza strains) | μg of anti-influenza HA antibodies/ml (CV [%]) | | |
| --- | --- | --- | --- |
|  | IgG | IgA | IgM |
| A/Cal09 | 130.8+14.6 (11.2) | 21.2+1.2(5.9) | 1.1+0.2(16.0) |
| A/Perth09 | 118.4+4.9 (4.3) | 1.4+0.1(9.6) | 0.7+0.1(14.6) |
| A/Vic09 | 140.7+6.6(4.7) | 1.8+1.0(37) | 0.9+0.2(25.8) |
| A/Vic11 | 161.6+4.6(2.8) | 2.6+0.3(10.6) | 1.6+0.4(23.2) |
| A/P.C73 | 34.1+3.6(10.5) | 0.8+0.2(18.9) | 0.4+0.1(23.6) |
| A/HK68 | 69.4+10.4 (15.0) | 3.1+0.5(17.1) | 0.9+0.2(23.2) |
| B/Bri08 | 359.5+71.9(20.0) | 10.1+0.9(8.7) | 1.4+0.1(8.3) |
| B/Wis10 | 335.2+29.6(8.8) | 11.43+1.1(9.2) | 1.1+0.2(21.6) |
